# Editorial: Single Cell Analysis – Discovery, Development and Implications to Study Cell-Cell and Cell-Pathogen Interactions

**DOI:** 10.3389/fcell.2022.951506

**Published:** 2022-06-22

**Authors:** Weimin Gao, Deepa Rana Jamwal, Jiangxin Wang, Hua Xu

**Affiliations:** ^1^ Barrow Neurological Institute (BNI), Phoenix, AZ, United States; ^2^ Department of Pediatrics, University of Arizona, Tucson, AZ, United States; ^3^ Shenzhen Key Laboratory of Marine Bioresource and Eco-Environmental Science, Shenzhen Engineering Laboratory for Marine Algal Biotechnology, Guangdong Provincial Key Laboratory for Plant Epigenetics, College of Life Sciences and Oceanography, Shenzhen University, Shenzhen, China

**Keywords:** single-cell, cell-pathogen interaction, intercelluar interaction, microfluidics, transcriptome profiling, multiplexed *in situ* protein analysis

**FIGURE 1:**
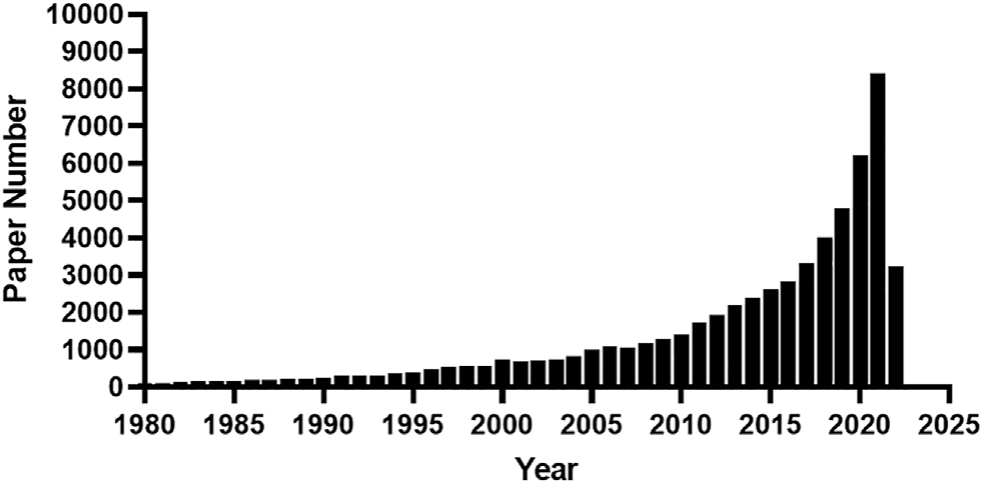
A counting statistics of published manuscripts devoted to single-cell analysis. The key word “single-cell “was used for searching the abstracts of published manuscripts from 1980 till May of 2022 in the PubMed database.

Like other scientific disciplines, the development of biology is driven mainly by technological developments. The invention of the microscopy in the seventeenth century led to the discovery of the cell and the establishment of the cell theory in the nineteenth century. The continued innovation in microscopy and related staining techniques have greatly advanced our understanding of cellular and subcellular structure and its composition till today. In addition to microscopy, scientists have also begun to develop other techniques for the analysis and manipulation of single cells since its discovery. Single colony isolation and culture, as the earliest approach for single-cell isolation and manipulation, has paved the way for pure culture cultivation since its invention in the nineteenth century and was first applied for single-celled organisms and then multicellular organisms. Howland and Bernstein (1931) explored a method for single-cell oxygen consumption in the 1930s, which is the prelude to its most recent method development (Kelbauskas et al., 2017). Since the 1970s, scientists have begun to explore methods for measuring multiple characteristics of a single neuron, so-called single-neuron recording techniques (Curthoys and Webber, 1977; Batuev et al., 1978). Also, the invention of fluorescence-activated cell sorting in the 1960s has dramatically facilitated the manipulation and study of single cells (Herzenberg et al., 2002). Since 2000, and especially the last decade, we have witnessed a rising publication wave of single-cell analysis (Figure 1). Currently, there are thousands of articles on single-cell analysis published each year. This is driven by the great development of techniques in relation to single-cell enabled techniques, collectively called single-cell omics, such as single-cell DNA sequencing, single-cell proteomics, etc. Instead of averaging signals from bulk cells, single-cell analysis has permitted us to reveal heterogeneity which existed even within isogeneic cell populations. Single-cell analysis has made and will be continuously making great progress in terms of both discovery of new cellular phenomena in various kinds of research areas and novel platform innovation. Based on single-cell analysis, new eukaryote cell types have been identified in immunological and neurological systems, and in cancerous tumors, etc., as demonstrated in the most recent gigantic efforts (Conde et al., 2022; Eraslan et al., 2022; Suo et al., 2022; The Tabula Sapiens Consortium, 2022). Without doubt, new biomarkers, novel diagnostic methods and treatments will be developed based on these discoveries.

The Research Topic we edited collects a total of eight manuscripts. As one droplet in the tide of single-cell analysis, these manuscripts contributed to and advanced the techniques for single-cell analysis, as well as our understanding of novel cellular properties and cell-cell interactions from a single-cell perspective.

Liao et al. reported a novel method for highly sensitive and multiplexed *in situ* protein analysis. Compared with other methods, this approach enhanced the detection sensitivity and reduced the imaging time by 1–2 orders of magnitude that potentially enable to detect hundreds of proteins within a single cell. Applying this method, the authors studied protein expression heterogeneity in a population of genetically identical cells and performed protein expression correlation analysis to identify co-regulated proteins. They then also profiled >6,000 neurons in a human formalin-fixed paraffin-embedded (FFPE) hippocampus tissue. By clustering these neurons into varied cell groups based on their multiplexed protein expression profiles, the authors observed different sub-regions of the hippocampus consisting of neurons from distinct cell groups.

Microfluidics-based technology has provided a powerful platform in approach development for single-cell analysis. Pan et al. provided an excellent review on the technical development of microfluidics-based single-cell research for intercellular interactions. This review focuses on microfluidic single-cell studies for intercellular interaction in a 2D or 3D environment with a variety of cell manipulating techniques and applications and the challenges to be overcome in the future.

Given the complexity of the tissues, it is never a trivial task to release single cells without damage and with least disturbance of physiological status from their matrixes for downstream manipulation and single-cell analysis. Here Yaigoub et al. provided a protocol to isolate viable single cells with high yield and purity from a human kidney tissue biopsy sample.

Although recent technical advancements have been first applied on mammalian cells, they also have potential application on prokaryotic cells, which are much smaller in size than that of eukaryotes. Chen et al. provided an excellent opinion article highlighting the recent progress on methods for single-cell analysis, especially on those currently only applying on mammalian cells but having potentials for microbial applications. The authors also presented perspectives on the future trends of technology development in the field of analyzing microbes at single-cell level.

Through the development of past 20 years, single cell sequencing, combining with follow-up bioinformatic analysis, has become the most sophisticated technological platform for single-cell analysis. In this Research Topic, three articles applied this technology to single-cell analysis. Through single-cell transcriptome profiling, Xiao et al. revealed the suppressive role of retinal neurons in microglia activation under diabetes mellitus. This study is the first to profile cell-specific molecular changes and the cell-cell interactome of retina under diabetes mellitus, paving the way to dissect the cellular and molecular mechanisms underlying early-stage diabetic retinopathy. Lin et al. unveiled the heterogeneity of nonimmune cells in chronic apical periodontitis. This study presented potential clues that inflammatory cytokines, chemokines, proteases, and growth factors initiated polymorphic cell differentiation, lymphangiogenesis, and angiogenesis during CAP. Wang et al. revealed the presence of complex gene expression alterations in human fetuses with trisomy 18 by applying single-cell transcriptomics of cultured amniotic fluid cells. This study indicated the complexity of trisomy 18 at the gene expression level. It revealed the genetic reasoning of diverse phenotypes in trisomy 18 patients.

Since its invention in the seventeenth century, microscopy and related staining techniques, have continually contributed to our understanding of cell and cell-cell interactions. In this Research Topic, Jones et al. have provided another example showing how insightful results could be reached if following this brilliant tradition. In this study, the authors used live-cell imaging of susceptible rice cells invaded by the pathogen cells of the hemibiotrophic fungus *Magnaporthe oryzae*, which express various fluorescent reporters, to investigate infection development in the invaded cells. The authors demonstrated that *M. oryzae* undergoes three distinct infection phases in the first-invaded cell before re-establishing biotrophy in the second-invaded cells. Understanding how infection phase-specific cellular dynamics are regulated and linked to host susceptibility will offer potential targets that can be exploited to control many plant diseases.
